# A scoping review on the links between sustainable development goal 14 and early childhood caries

**DOI:** 10.1186/s12903-023-03650-3

**Published:** 2023-11-18

**Authors:** Morenike Oluwatoyin Folayan, Imen Ayouni, Arthemon Nguweneza, Ola Barakat Al-Batayneh, Jorma I. Virtanen, Balgis Gaffar, Duangporn Duangthip, Ivy Guo Fang Sun, Nneka Kate Onyejaka, Hamideh Daryanavard, Tshepiso Mfolo, Carlos A. Feldens, Robert J. Schroth, Maha El Tantawi

**Affiliations:** 1 Early Childhood Caries Advocacy Group, Ile-Ife, Nigeria; 2 Department of Child Dental Health, Obafemi Awolowo University, Ile-Ife, Nigeria; 3 Nigeria Institute of Medical Research, Yaba, Lagos, Nigeria; 4 Department of Pediatrics and Child Health, Faculty of Health Sciences, University of Cape Town, Cape Town, South Africa; 5 Division of Human Genetics, Department of Pathology, Faculty of Health Sciences, University of Cape Town, Cape Town, South Africa; 6 Preventive Dentistry Department, Jordan University of Science and Technology, Irbid, Jordan; 7 Faculty of Medicine, University of Bergen, Bergen, Norway; 8 Department of Preventive Dental Sciences, College of Dentistry, Imam Abdulrahman bin Faisal University, Dammam, Saudi Arabia; 9 Faculty of Dentistry, The University of Hong Kong, Hong Kong SAR, China; 10 Department of Child Dental Health, Faculty of Dentistry, University of Nigeria, Enugu Campus, South Africa; 11 Dubai Health Authority, Dubai, United Arab Emirates; 12 University of Pretoria, Pretoria, South Africa; 13 Department of Pediatric Dentistry, Universidade Luterana Do Brasil, Canoas, Brazil; 14 Dr. Gerald Niznick College of Dentistry, University of Manitoba, Winnipeg, Canada; 15 Department of Pediatric Dentistry and Dental Public Health, Faculty of Dentistry, Alexandria University, Alexandria, Egypt

**Keywords:** Sustainable development, Oceans and seas, Hydrobiology, Child, Preschool, Dental caries, Marine life, Ecosystem

## Abstract

**Background:**

The Sustainable Development Goal (SDG) 14 addresses life below the waters, an important source of protein and contributor to global food security and economic development. Our aim was to explore possible evidence on the links between life below water and early childhood caries (ECC).

**Methods:**

This scoping review identified articles on the link between life below water and caries according to the PRISMA-ScR guidelines. Three electronic databases (PubMed, Web of Science, and Scopus) were systematically searched in January 2023, using specific search terms. Studies written in English, with full text available, addressing life under water, focusing on dental caries in humans, with results that can be extrapolated to control ECC in children less than 6 years of age were included in the review. Descriptive statistics were used to summarize the retrieved papers and graphical presentation was used for visualization.

**Results:**

There were 224 publications retrieved of which 13 studies, published between 1960 and 2022, were included in the analysis. The papers originated from Asia (7/13), North America (3/13), Europe (1/13), and 2/13 had multi-country authorship. Also, four laboratory studies extracted agents from marine products to determine their efficacy in preventing caries formation and preventing/slowing plaque formation; four letters discussed the caries prevention potential of sea salt as a source of fluoride; and two review articles about the positive effects of extracted marine products for caries prevention. Most (11/13) studies addressed target 14.1 concerned with enriching the marine environment with nutrients and minerals; two addressed target 14.4 focused on ensuring fish stocks are within biologically sustainable levels; two addressed target 14.7 aimed at increasing the economic benefits through sustainable use of marine resources such as fisheries; and one focused on target 14.5 aimed at conserving marine areas by increasing protected areas. In addition, one ecological study assessed the association between the ecosystem and ECC.

**Conclusions:**

Currently, there is little known about the impact of protection of marine and coastal ecosystem from pollution and ocean acidification on the risk of ECC. Further evidence on possible associations between life below water and ECC management is needed.

**Supplementary Information:**

The online version contains supplementary material available at 10.1186/s12903-023-03650-3.

## Introduction

The Sustainable Development Goal 14 (SDG14) has multiple objectives centered around promoting the sustainability and well-being of marine ecosystems. These goals encompass various aspects such as reducing marine pollution, safeguarding and restoring ecosystems, mitigating ocean acidification, ensuring sustainable fishing practices, conserving coastal and marine areas, eliminating subsidies that contribute to overfishing, maximizing economic benefits from the sustainable utilization of marine resources, advancing scientific knowledge and research for ocean health, supporting small-scale fishers, and enforcing international maritime laws [1]. SDG14 comprises 10 specific targets labeled as 14.1 to 14.7, 14.a, 14.b, and 14.c. Furthermore, there are 10 indicators associated with these targets. These indicators range from measuring the index of coastal eutrophication and plastic debris density (14.1.1) to assessing the progress made by countries in ratifying, accepting, and implementing legal, policy, and institutional frameworks related to ocean-related instruments that align with international law outlined in the United Nations Convention on the Law of the Sea. These efforts aim to ensure the conservation and sustainable utilization of oceans and their resources [2].

The Earth’s oceans, which cover 71% of the planet’s surface, play a significant role in providing animal protein, accounting for approximately 17% of the world’s per capita consumption. However, in terms of caloric food supply, they contribute only 2% [3, 4]. For thousands of years, the oceans and seas have served as vital sources of protein [5], contributing to food security and economic development in numerous nations [6]. Fish alone provide at least 20% of the daily animal protein intake for over 3.3 billion people worldwide [7]. The vast biodiversity and undiscovered species within the oceans offer potential for the development of new oral health products and innovative compounds to combat diseases [8]. Communities that rely on marine diets have been found to have lower rates of dental caries [9, 10]. In 2017, these communities represented approximately 37% of the global population [11]. Therefore, incorporating seafood into diets as an alternative to animal protein [12, 13] could potentially contribute to addressing the worldwide burden of dental caries.

The oceans and coastal areas face significant vulnerabilities due to environmental degradation, overfishing, climate change, and pollution [14]. Ocean acidification has adverse effects on human access to food, oxygen, livelihoods, blue spaces, and medicines [14–19]. This, in turn, increases the risks of malnutrition, poisoning, respiratory diseases, poor mental health, and the depletion of medicinal resources [20, 21]. Approximately 300 million people who depend on the ocean for food security and livelihoods may be impacted by this acidification [22]. Preserving the ocean’s ecosystem not only supports the development of products that can hinder the activities of organisms associated with the formation of tooth decay and slow down the formation of dental plaque, thereby reducing the risk of caries [23], but it also serves as a valuable source of animal protein that promotes food security [6]. The preservation of ocean ecosystems can contribute to the prevention of early childhood caries (ECC) by reducing the risk of malnutrition in children, [24]. This is because malnutrition is a risk factor for ECC [25, 26].

ECC is as a global public health concern, affecting more than 514 million children worldwide [27]. ECC is defined as the presence of both cavitated and non-cavitated tooth decay on one or more primary teeth in children under 72 months of age [28]. If left untreated, ECC can result in pain, infection, contribute to malnutrition, hinder psychological development and physical growth, reduce the overall quality of life related to oral health, and in rare cases, even lead to fatalities [28–32]. The impact of the environment on the food chain can exacerbate malnutrition. The inter-link between the impact of the environment on food chain and the resultant malnutrition, leading to a higher prevalence of caries in vulnerable marine communities, particularly those in low and low-middle income countries [32–35].

The association between dental caries and SDG14 (life below water) remains largely unexplored. However, it is plausible to consider that the negative impacts on the ocean and life below water could potentially elevate the risk of ECC. The objective of this scoping review was to examine existing evidence regarding the connection between life below water and the prevention and treatment of dental caries. Furthermore, the review aimed to identify potential links between life below water and ECC.

## Methods

A search was performed to identify published literature in peer-reviewed journals on the association between life below water and ECC. Our scoping review was conducted in accordance with the Preferred Reporting Items for Systematic Reviews and Meta-Analyses Extension for Scoping Reviews guidelines (PRISMA-ScR) [36]. This review was guided by the questions: (i) What is the existing evidence on the possible links between life below water and caries? and (ii) what are the possible links between life below water and ECC?

### Search strategy

The initial search was conducted on three electronic databases in January 2023. The databases were PubMed, Web of Science, and Scopus. The search terms and strategies used for each database are listed in Appendix 1.

### Literature search and inclusion

Publications identified through the search strategy were exported to the reference management software Mendeley (version 1.19.8) and duplicates were removed. Title and abstract screening were performed independently by two researchers (AN and AI) using pre-defined inclusion and exclusion criteria. Full-text review of the remaining publications was then completed independently by four researchers (AN, AI, MOF and MET) and reference lists of potentially relevant publications were manually searched. Uncertainty regarding whether publications met the inclusion criteria was resolved via consensus among the three researchers.

### Inclusion criteria

Articles were considered for inclusion if they were written in English, with full text available, addressing an organism or component of life under water and focusing on dental caries. There was no restriction by type of article, study design or time of publication.

### Exclusion criteria

To answer the research question on the links between life below water and ECC, we excluded papers without data on children < 72 months of age or papers explicitly excluding this age group. Other types of data sources such as websites or books were excluded.

### Data extraction

A data extraction form was developed and pilot tested. In addition to the paper identifiers (title, author, link), the following data were extracted: country, year of publication, study design, life under water component included, whether published in dental or non-dental journals. Information on the study objectives and the conclusion reached were also extracted.

### Role of the funding source

The present study was funded by out-of-pocket expenses. This had no role to play in the study design, data collection and analysis, decision to publish, or preparation of the manuscript.

### Analysis

Descriptive statistics as frequencies and percentages were used to summarize the categories of retrieved papers and graphical presentation was used for visualization. An analysis was conducted to identify the countries of origin of the manuscripts, the caries prevention or treatment focuses of the publication, studies that reported associations between life below waters and ECC, and the SDG14 indicators the extracted studies addressed.

## Results

**Fig. 1:**
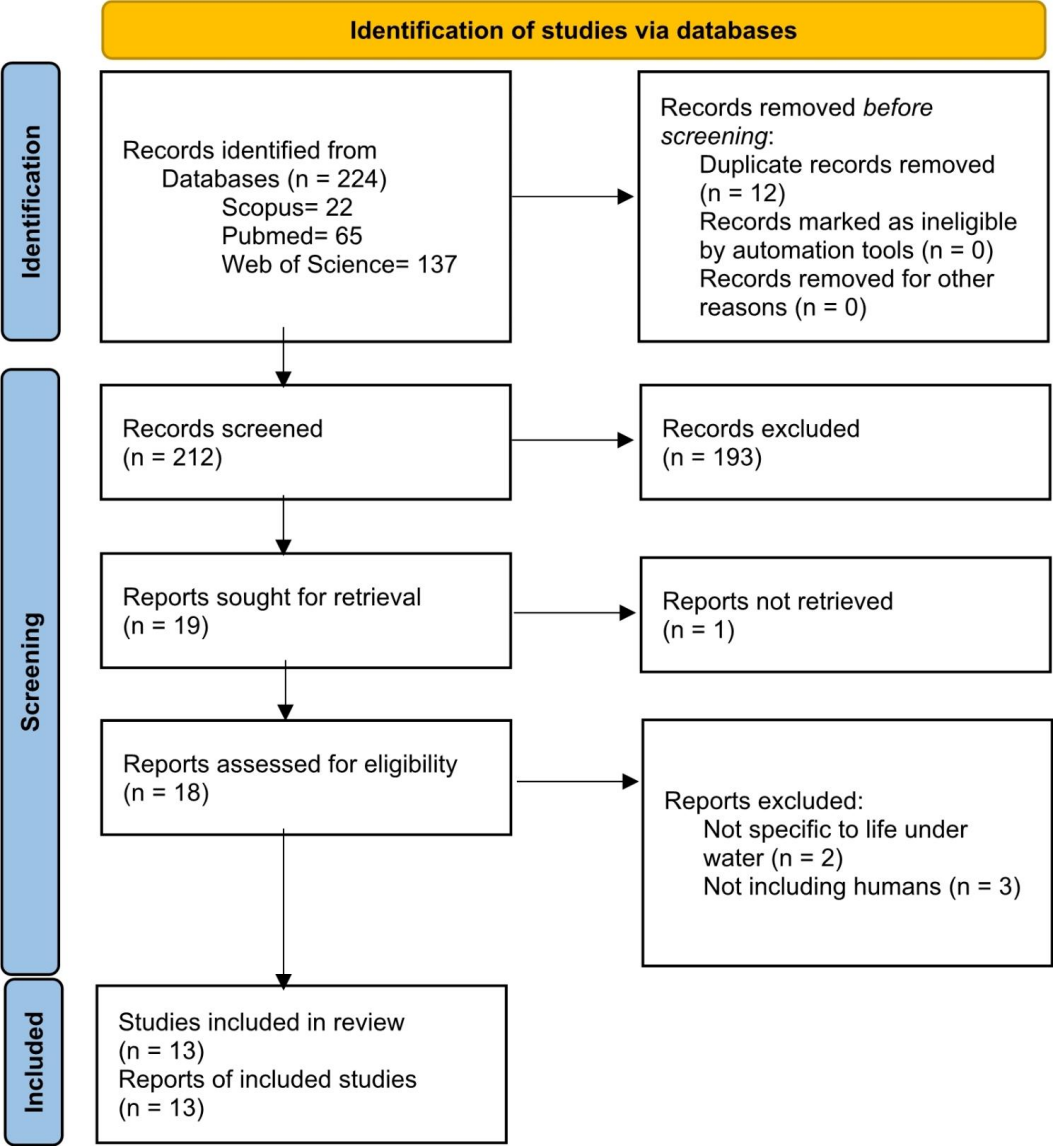
Resources included in the scoping review based on the PRISMA 2020 method [36]

Figure 1 shows the process undertaken to identify relevant literature. The initial search across three databases resulted in 224 potentially relevant publications. After eliminating 12 duplicate papers, a total of 212 papers underwent screening based on their titles, abstracts, and full texts. Among these, 199 papers were excluded as they did not meet the eligibility criteria. Reasons for exclusion included being unrelated to life underwater, lacking human participants, focusing solely on individuals over 71 months of age, not addressing caries, being non-English publications, or lacking full-text availability. Out of the remaining papers, 13 studies [36–49] provided data on potential connections between life below water and caries. However, only one ecological study was found that reported an association between life below water and ECC [38]. Table 1 presents further details regarding the 13 included publications.

**Fig. 2:**
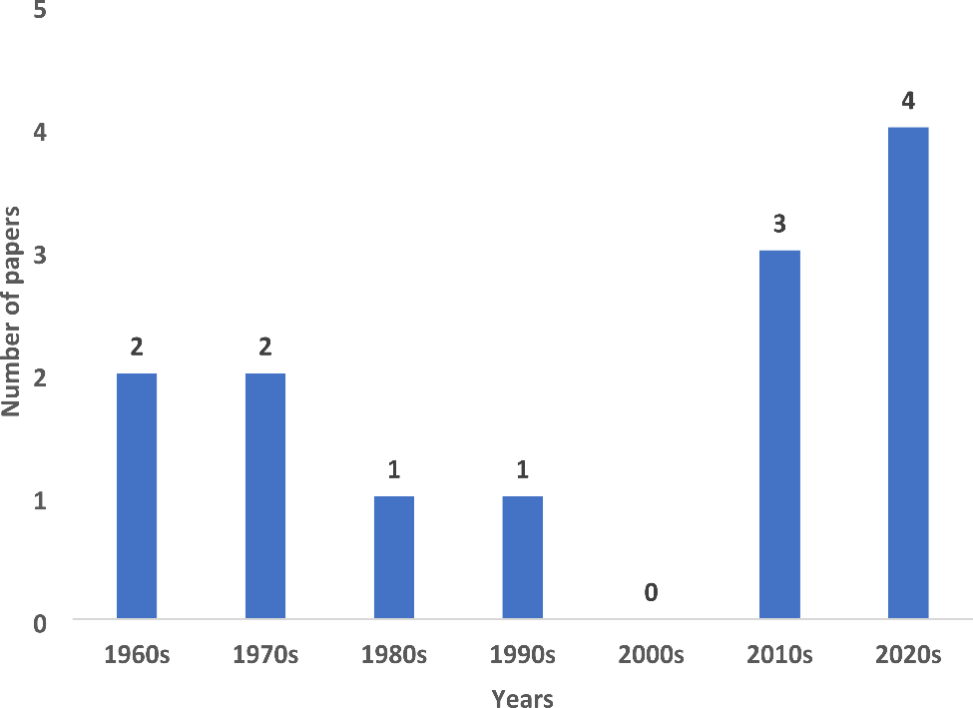
Number of papers on SDG14 related elements and ECC over time

Figure 2 shows that the two papers on the link between life under water and caries were published in the 1960s [46, 47]. Between 1970 and 2000, four papers were published [39, 40, 44, 45]; and from 2000 to 2022, seven additional papers were published [36, 37, 41–43, 47, 48].

Out of the thirteen papers analyzed, seven originated from Asian countries, including China [41–43, 48], India [37, 44], and Japan [39]. Three papers were from the United States [44–47], and one paper was from Europe, specifically Norway [40]. Two papers had authors from multiple countries [38, 48]. Among the thirteen papers, two were published in dental journals, namely Caries Research and Scandinavian Journal of Dental Research [39, 40]. In addition, four laboratory studies were identified. These studies focused on extracting substances from marine products, such as marine bacteria and seaweed, to evaluate their effectiveness in preventing caries formation [39, 41, 42], as well as preventing or slowing down plaque formation [43]. Additionally, a fifth laboratory study compared the rate of dissolution between fluoroapatite (shark enamel) and hydroxyapatite (human enamel) when exposed to a high caries challenge [40].

**Table 1:** Characteristics of the studies included in the scoping review (n = 13)

Author (Publication year)	Location	Study design	SDG14 goal	Study focus	Study objective	Conclusions
Asawa et al., 2014. [37]	India	Cross-sectional	14.7.1	Workplace environment	Assess and compare the oral health status of fishermen and non-fishermen population of Kutch District, Gujarat, India	Fishermen population showed significantly greater proportion of persons with dental caries (82.6%) than non-fishermen population (44.6%) (p = 0.001)
*Folayan et al. 2020. [38]	Multi-country	Ecological	14.1.1 14.4.1 14.5.1 14.7.1	Marine protected areas, fish stock status, regional marine trophic index	Determine the association between 24 global environmental indicators and ECC in 3-5-year-old children.	Of the 24 environmental indicators, eight had at least a small-effect size but non-significant association with ECC in 3–5-year-old children: percentage of marine protected areas (ƞ2 = 0.03), species habitat index (ƞ2 = 0.06), percentage of tree cover loss (ƞ2 = 0.03), regional marine trophic index (ƞ2 = 0.03), total carbon dioxide emission intensity (ƞ2 = 0.03), methane emission intensity (ƞ2 = 0.04), nitrous oxide emission intensity (ƞ2 = 0.06), and sulfur dioxide emission intensity (ƞ2 = 0.03).
Saeki et al., 1996. [39]	Japan	Laboratory	14.1.1	Seaweed	Examine the effect of funoran on the absorption of oral streptococci to saliva-coated hydroxyapatite in vitro and its anticariogenic on experimental rats infected with Streptococcus sanguis.	The colonization of S.sobrinus 6715 inoculated on the molar teeth of experimental rats that were administered funoran was less frequent than that in a funoran-free group. The mean buccal and lingual, sulcal, and total caries scores of rat groups administered funoran (a sulfated polysaccharide extracted from the seaweed Gloiopeltis furcate) were significantly lower than those of the funoran-free group.
Ogaard et al. 1988. [40]	Norway	Laboratory	14.1.1	Shark teeth	Compare the resistance of fluoroapatite (shark enamel) and hydroxyapatite (human enamel) against a high caries challenge in a human in vivo model	The mean total mineral loss (delta Z) was 1680 vol% micron in human enamel and 965 vol% micron in shark enamel. The corresponding mean values for lesion depth were 90 micron and 36 micron respectively.
Ren et al. 2018. [41]	China	Laboratory	14.1.1	Marine bacterium	Evaluate the ability of dextranase from a marine bacterium *Catenovulum* sp. (Cadex) to impede formation of *Streptococcus mutans* biofilms,	Cadex was shown to be an alkaline and cold-adapted endo-type dextranase that impeded the formation of *S. mutans* biofilm to some extent, and suitable for development of a novel marine agent for the treatment of dental caries
Xu et al., 2022. [42]	China	Laboratory	14.1.1	Marine bacterium	Identify and characterize the enzymatic properties, hydrolysis characteristics, protein sequence and 3D structure of CeDex and its effect on suppressing and removing dental plaque.	CeDex (a dextranase from the marine bacterium *Cellulosimicrobium* sp. THNI could prevent the formation of *Streptococcus mutans* biofilm and disassemble existing biofilms at 10 U/ml concentration
Jiao et al. 2014. [43]	China	Laboratory	14.1.1	Marine bacterium	To purify and characterize a dextranase (Dex410) from marine Arthrobacter sp. and compare this with fungi derived dextranase containing commercial mouthwashes	For short-term use (1.5 months), both Dex410 and the commercial mouthwash Biotene (Laclede Professional Products, Gardena, CA, USA) had a significant inhibitory effect on caries (p = 0.0008 and 0.0001, respectively), while for long-term use (3 months), only Dex410 showed significant inhibitory effect on dental caries (p = 0.005).
*Rao,1971 [44]	India	Letter	14.1.1	Sea salt	-	Sea salt contributes fluoride to the amount of 0.05 to 0.34 mg/day and is not enough to contribute fluoride to the human diets in India to give protection against caries.
Hadjimarkos, 1972 [45]	USA	Letter	14.1.1	Sea salt	-	In countries where the consumption of salt is high because of local dietary habits and food customs, the use of crude sea salt would make a significant contribution to fluoride intake
Hadjimarkos, 1962 [46]	USA	Letter	14.1.1	Sea salt	-	Serious consideration should be given to the role of sea salt as an important source of dietary fluoride for the prevention of dental caries in areas of the world where the salt consumed locally is prepared by evaporating sea water.
Hadjimarkos, 1965 [47]	USA	Letter	14.4.1	Fish flour	-	Selenium increases the susceptibility of teeth to dental caries. Fish flour, which is being increasingly used as a food supplement for the prevention and treatment of protein malnutrition, is one of the foods with the highest content of selenium.
Barzkar, 2022 [48]	Multi-country	Review	14.1.1	Marine bacterium	A review of the properties of dextran, properties of dextran-hydrolyzing enzymes, particularly from marine sources, the biochemical features of these enzymes and the potential use of marine bacterial dextranase to remove dental plaque.	Dextranase from marine bacteria is the most preferable for removing plaque, as it has a high enzymatic activity.
Huang et al., 2021 [49]	China	Review	14.1.1	Marine bioactive compounds	Overview of different marine-sourced bioactive compounds and their health benefits in dental caries, gingivitis, periodontitis, halitosis, oral cancer, and their potential use as functional food ingredients for oral health	Marine bioactive ingredients seaweed extracts, n-3 PUFAs, sea cucumber extracts, and marine bacterial metabolites have the ability to inhibit oral pathogens, repress their biofilms, and regulate the cancer cell cycle.

* Negative results

The letters to the editor, written during the 1960 and 1970 s, focused on the potential of sea salt as a source of fluoride for caries prevention [43–46], as well as the cariogenic potential of fish flour due to its selenium content [47]. However, Rao [44] found that the fluoride content in the salt was not sufficient to have anticariogenic effects. Two review articles discussed the positive effects of extracted marine products in preventing caries [48, 49]. Additionally, there were two observational studies: one ecological study [38] and one cross-sectional study [37], examining caries risk indicators. No cohort studies or clinical trials were identified.

The most investigated area was the use of extracted compounds from marine sources for caries prevention, including marine bacteria [41–43, 47], bioactive compounds [49], and seaweed [39]. Other areas of research focused on sea salt [43–46], fish products such as fish flour [47], and shark teeth [40]. The observational studies included caries risk indicators related to the workplace environment [37] and environmental indicators such as marine protected areas, fish stock status, and the regional marine trophic index [38].

Table 1 demonstrates that most studies (11 out of 13) addressed SDG14 target 14.1, which aims to enrich the marine environment with nutrients and minerals. Two studies focused on target 14.4, aiming to ensure fish stocks are maintained at sustainable levels. Two studies examined target 14.7, aiming to increase economic benefits through the sustainable use of marine resources, particularly fisheries. One study addressed target 14.5, which involves conserving marine areas by expanding protected areas. Each study focused on one target, except for one study [38], which addressed four targets.

**Fig. 3:**
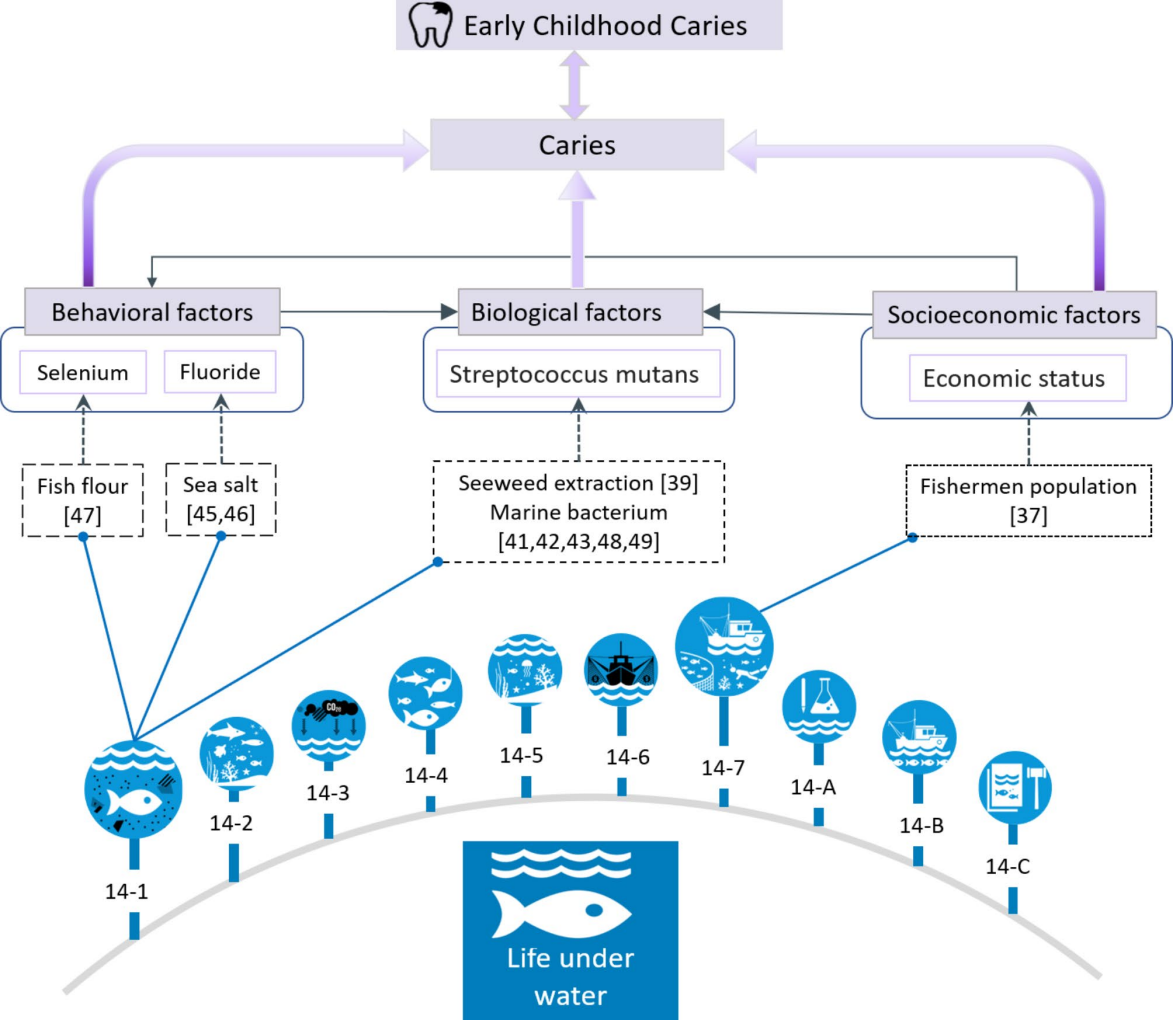
The conceptual framework of early childhood caries and life under water (SDG14) indicating the publications, and the mechanism by which they are linked with behavioural, biological and socioeconomical risk factors for caries. These links to caries can also be linked to early childhood caries 14 − 1 Reduce marine pollution 14 − 2 Protect and restore ecosystems 14 − 3 Reduce Ocean acidification 14 − 4 Sustainable fishing 14 − 5 Conserve coastal and marine areas 14 − 6 End subsidies contributing to overfishing 14 − 7 Increase the economic benefits from sustainable use of marine resources 14-A Increase scientific knowledge, research, and technology for ocean health 14-B Support small scale fishers 14-C Implement and enforce international sea law

Figure 3 presents our proposed conceptual framework linking ECC and life under water. The evidence suggests that the connection between SDG14 and ECC can occur through its impact on the epidemiological profile of ECC or by moderating ECC risk factors. The relevant SDG14 targets linked to ECC are 14.1 and 14.7. Products derived from the oceans can enhance fluoride availability in the oral cavity or reduce the activity of Streptococcus mutans, thereby reducing tooth susceptibility to ECC. Conversely, ocean acidification and belonging to coastal indigenous communities, such as fishermen populations, are associated with a higher risk of ECC.

## Discussion

While our study postulates a potential link between life below water and ECC, no direct evidence supporting an association between SDG14, caries, and ECC was found. However, evidence from basic science studies suggests the use of ocean-derived substances to reduce caries risk through the production of dental products that inhibit dental bacterial biofilm formation, plaque retention, and through food sources rich in fluoride.

A scoping review was performed to survey the body of literature concerning the correlation between two significantly vital public health concerns and to identify gaps in knowledge [50]. This approach aids in determining which specific SDG14 targets can be advanced to a systematic review and which ones necessitate ecological analysis to generate additional evidence. One of highlights of the study is the need for ecological studies to generate preliminary evidence on the association between targets of the SDG 14 and ECC.

In addition, the findings of this scoping review suggest that effective management of the world’s oceans could counterbalance the detrimental effects of climate change on oral health [51]. Currently, over 30% of the world’s fish stocks are overexploited [52], and ocean pollution is a global issue that destroys marine ecosystems [53] and affects atmospheric oxygen production [54]. By promoting the sustainable utilization of oceans and adopting seafood-based diets [55], it is hypothesized that the risk of ECC could be reduced, leading to a decline in its global prevalence.

Most seafood naturally contains fluoride due to the presence of sodium fluoride in the ocean [56], and fluoride is known to reduce caries risk [57]. Sea salts, for example, can contain as high as 40 ppm of fluoride [58]. Additionally, fish and other seafood are rich sources of omega-3 fatty acids [59], which are associated with a reduced risk of active caries [60]. Coastal groundwater, which is often consumed by coastal communities, is also rich in calcium and associated with a low prevalence of caries [61]. The consumption of seaweeds, which is increasing in popularity [62], also provides varying amounts of fluoride based on species, environmental characteristics, water temperature, and nutrient content of seawater [63, 64]. Furthermore, bioactive compounds sourced from the ocean have the potential to serve as functional food ingredients for caries prevention [65].

However, the reality of caries risk is complex, and multiple factors contribute to its prevalence. Coastal indigenous communities, who heavily rely on seafood, often exhibit high ECC prevalence rates. For example, in an Indian coastal community, ECC prevalence was as high as 75% in children aged 0–4 years with an average of 4.68 decayed, filled teeth (dft), and 86.45% in children aged 5–9 years with an average dft of 5.09 [66]. Another Indian coastal community reported a caries prevalence of 5.11% in children aged 5–8 years [67]. These findings indicate that living in coastal areas alone, without considering broader preventive oral health behaviors, food habits, lifestyle, and other cultural and socioeconomic contexts, may not necessarily result in low ECC prevalence. Many coastal communities face high levels of poverty, limited access to oral health services, low oral health awareness, and habits such as infrequent toothbrushing and high consumption of sugary foods [68, 69]. Therefore, future studies should consider conducting comparative evaluations of marine-dependent communities while controlling for possible confounders.

As shown in Table 2, the profile of ECC prevalence in countries with high seafood consumption does not consistently demonstrate a trend toward low caries prevalence. This finding suggests that country-level factors may influence caries risk in primary dentition. For instance, Japan has achieved significant improvements in oral health over the years through its oral health system [70], while China has faced neglect in oral health care [71], and countries like Myanmar, India, Vietnam, and Malaysia have various challenges in terms of investment, policy implementation, and equitable access to public healthcare [71–75]. Consequently, SDG14 may complement the efforts of other SDGs to have a measurable impact on ECC prevalence through the development of data-driven strategies based on legislation, policies, and technology [76].

**Table 2:** Countries that eat the most fish (tonne per person) and the prevalence of ECC

Rank	Country	Tonnes of fish consumed per person (tonnes consumed per country*/population of country**)	ECC prevalence (%) in children aged < 36 months***	ECC prevalence (%) in children aged 36–71 months***
1	Myanmar	0.028	Data before 2007	50.0
2	Vietnam	0.011	No data	73.7
3	Malaysia	0.010	No data	98.1
4	Japan	0.006	3.9	23.9
5	Cote d’Ivoire	0.006	No data	No data
6	Mozambique	0.004	No data	No data
7	Mexico	0.002	34.0	61.5
8	China	0.001	8.8	62.2
9	Indonesia	0.0005	35.8	79.5
10	India	0.0003	38.9	52.1

* WorldAtlas [77]; **The World Bank [78]. ***El Tantawi et al. [79]

Table 2 also highlights the high consumption of fish in Asia, with seven out of the top ten fish-consuming nations located in the region. Similarly, a significant number of publications on caries and SDG14 originate from Asia. Despite the potential benefits that oceanic products offer in terms of anti-cariogenic properties, ECC prevalence remains extremely high in this region, particularly in Southeast Asia [24, 79]. The study by Folayan et al. [38], however, suggests that the ocean alone may not be sufficient in reducing ECC, as the associations between marine ecosystem variables and ECC were not significant, and the effect size was small. Rao [44] also indicates that the fluoride content in sea salt may not provide adequate protection against caries. Therefore, future studies should aim to comprehend how multiple factors may limit the effectiveness of oceanic resources in reducing caries risk, including ECC.

Furthermore, ocean acidification, which leads to airway irritants and discomfort, can worsen asthma symptoms, and decrease pulmonary function [80]. Respiratory disorders directly increase the risk of ECC [81, 82], or indirectly through a higher likelihood of developing enamel defects [83, 84], often caused using asthma medications [85]. Additionally, the loss of livelihood due to the collapse of the fishing industry may diminish the fundamental “nature-connectedness” that promotes mental health [85–88]. Poor mental health is associated with a higher risk of caries, partly due to neglecting oral hygiene and partly due to xerostomia resulting from antidepressant use [89]. Similarly, parental mental health disorders are linked to an increased ECC risk in preschool children [90].

This scoping review has some limitations. Firstly, it did not include literature from databases not covered in the review or studies published in languages other than English. This may have resulted in an underestimation of publications examining the links between SDG14, caries, and ECC. Additionally, we did not explore the existence of dental products currently available in the market that utilize underwater components to control and prevent ECC. This is an area that could be explored in future studies. Despite these limitations, this scoping review is the first to provide an overview of research on the link between SDG14 and ECC, identifying research gaps and emphasizing the need to explore the interconnection between SDG14 and other caries prevention strategies to mitigate the risk of ECC.

## Conclusion

In conclusion, our study highlights the limited knowledge regarding the impact of protecting marine and coastal ecosystems from pollution and ocean acidification on the risk of ECC. Further evidence is required to understand the potential association between bodies of water, marine life, and the global prevalence of ECC, as well as to develop effective ECC management strategies.

### Electronic supplementary material

Below is the link to the electronic supplementary material.

Supplementary Material 1

## Data Availability

All data generated or analysed during this study are included in this published article.

## Abreviations

CI: Confidence Interval
ECC: Early Childhood Caries
dft: decayed, and filled primary teeth
PRISMA-ScR: Preferred Reporting Items for Systematic Reviews and Meta-Analyses Extension for Scoping Reviews guidelines
PUFA: Pain Ulcer Fistula Abscess
SDG: Sustainable Development Goal
