# Correction: A scoping review on the links between sustainable development goal 14 and early childhood caries

**DOI:** 10.1186/s12903-023-03748-8

**Published:** 2024-01-10

**Authors:** Morenike Oluwatoyin Folayan, Imen Ayouni, Arthemon Nguweneza, Ola B. Al-Batayneh, Jorma I. Virtanen, Balgis Gaffar, Duangporn Duangthip, Ivy Guo Fang Sun, Nneka Kate Onyejaka, Hamideh Daryanavard, Tshepiso Mfolo, Carlos A. Feldens, Robert J. Schroth, Maha El Tantawi

**Affiliations:** 1 Early Childhood Caries Advocacy Group, Ile-Ife, Nigeria; 2 Department of Child Dental Health, Obafemi Awolowo University, Ile-Ife, Nigeria; 3 Nigeria Institute of Medical Research, Yaba, Lagos, Nigeria; 4 Department of Pediatrics and Child Health, Faculty of Health Sciences, University of Cape Town, Cape Town, South Africa; 5 Division of Human Genetics, Department of Pathology, Faculty of Health Sciences, University of Cape Town, Cape Town, South Africa; 6 Department of Orthodontics, Pediatric and Community Dentistry, College of Dental Medicine, University of Sharjah, Sharjah, PO Box 27272 United Arab Emirates; 7 Preventive Dentistry Department, Jordan University of Science and Technology, PO Box 3030, Irbid, Jordan; 8 Faculty of Medicine, University of Bergen, Bergen, Norway; 9 Department of Preventive Dental Sciences, College of Dentistry, Imam Abdulrahman Bin Faisal University, Dammam, Saudi Arabia; 10 Faculty of Dentistry, The University of Hong Kong, Hong Kong SAR, China; 11 Department of Child Dental Health, Faculty of Dentistry, University of Nigeria, Enugu Campus, South Africa; 12 Dubai Health Authority, Dubai, United Arab Emirates; 13 University of Pretoria, Pretoria, South Africa; 14 Department of Pediatric Dentistry, Universidade Luterana Do Brasil, Canoas, Brazil; 15 Dr. Gerald Niznick College of Dentistry, University of Manitoba, Winnipeg, Canada; 16 Department of Pediatric Dentistry and Dental Public Health, Faculty of Dentistry, Alexandria University, Alexandria, Egypt

**Correction: BMC Oral Health 23, 881 (2023)**

**https://doi.org/10.1186/s12903-023-03650-3**

In this article [1], the affiliation details for Prof. Ola B. Al-Batayneh were incorrectly given as “1 and 6” only (1. Early Childhood Caries Advocacy Group and 6. Preventive Dentistry Department, Jordan University of Science and Technology, Irbid, Jordan) but should have been “1, 6 and 7” (1. Early Childhood Caries Advocacy Group, and 6. Department of Orthodontics, Pediatric and Community Dentistry, College of Dental Medicine, University of Sharjah, Sharjah, PO Box 27272, United Arab Emirates and 7. Preventive Dentistry Department, Jordan University of Science and Technology, Irbid, Jordan).
