# Supplementary material for: A scoping review on the links between sustainable development goal 14 and early childhood caries
Source: BMC Oral Health. 2023 Nov 18;23:881. doi: 10.1186/s12903-023-03650-3 (PMC10657611; doi:10.1186/s12903-023-03650-3)
Supplement: Supplementary file 1 — Supplementary Material 1 [file 12903_2023_3650_MOESM1_ESM.docx]

Appendix 1: Search strategy in Pubmed, Scopus and Web of Science

PubMed

| # | Query | Search Details | Results |
| --- | --- | --- | --- |
| 4 | ("Dental Caries"[Mesh]) AND (("Fishes"[Mesh]) OR ("Marine Biology"[Mesh]) OR ("Eutrophication"[Mesh]) OR ("Seawater"[Mesh]) OR ("Seaweed"[Mesh]) OR ("Fisheries"[Mesh]) OR sea OR ocean OR underwater OR "plastic debris") | "Dental Caries"[MeSH Terms] AND ("Fishes"[MeSH Terms] OR "Marine Biology"[MeSH Terms] OR "Eutrophication"[MeSH Terms] OR "Seawater"[MeSH Terms] OR "Seaweed"[MeSH Terms] OR "Fisheries"[MeSH Terms] OR ("spat econ anal"[Journal] OR "sea"[All Fields]) OR ("ocean"[All Fields] OR "ocean s"[All Fields] OR "oceanic"[All Fields] OR "oceans and seas"[MeSH Terms] OR ("oceans"[All Fields] AND "seas"[All Fields]) OR "oceans and seas"[All Fields] OR "oceans"[All Fields]) OR "underwater"[All Fields] OR "plastic debris"[All Fields]) | 65 |
| 3 | ("Dental Caries"[Mesh]) AND (("Marine Biology"[Mesh]) OR ("Eutrophication"[Mesh]) OR ("Seawater"[Mesh]) OR ("Seaweed"[Mesh]) OR ("Fisheries"[Mesh]) OR sea OR ocean OR underwater OR "plastic debris") | "Dental Caries"[MeSH Terms] AND ("Marine Biology"[MeSH Terms] OR "Eutrophication"[MeSH Terms] OR "Seawater"[MeSH Terms] OR "Seaweed"[MeSH Terms] OR "Fisheries"[MeSH Terms] OR ("spat econ anal"[Journal] OR "sea"[All Fields]) OR ("ocean"[All Fields] OR "ocean s"[All Fields] OR "oceanic"[All Fields] OR "oceans and seas"[MeSH Terms] OR ("oceans"[All Fields] AND "seas"[All Fields]) OR "oceans and seas"[All Fields] OR "oceans"[All Fields]) OR "underwater"[All Fields] OR "plastic debris"[All Fields]) | 51 |
| 2 | ("Dental Caries"[Mesh]) AND (("Eutrophication"[Mesh]) OR ("Seawater"[Mesh]) OR ("Seaweed"[Mesh]) OR ("Fisheries"[Mesh]) OR sea OR ocean OR underwater OR "plastic debris") | "Dental Caries"[MeSH Terms] AND ("Eutrophication"[MeSH Terms] OR "Seawater"[MeSH Terms] OR "Seaweed"[MeSH Terms] OR "Fisheries"[MeSH Terms] OR ("spat econ anal"[Journal] OR "sea"[All Fields]) OR ("ocean"[All Fields] OR "ocean s"[All Fields] OR "oceanic"[All Fields] OR "oceans and seas"[MeSH Terms] OR ("oceans"[All Fields] AND "seas"[All Fields]) OR "oceans and seas"[All Fields] OR "oceans"[All Fields]) OR "underwater"[All Fields] OR "plastic debris"[All Fields]) | 51 |
| 1 | ("Dental Caries"[Mesh]) AND (("Eutrophication"[Mesh]) OR ("Seawater"[Mesh]) OR ("Seaweed"[Mesh]) OR ("Fisheries"[Mesh]) OR sea OR ocean OR underwater) | "Dental Caries"[MeSH Terms] AND ("Eutrophication"[MeSH Terms] OR "Seawater"[MeSH Terms] OR "Seaweed"[MeSH Terms] OR "Fisheries"[MeSH Terms] OR ("spat econ anal"[Journal] OR "sea"[All Fields]) OR ("ocean"[All Fields] OR "ocean s"[All Fields] OR "oceanic"[All Fields] OR "oceans and seas"[MeSH Terms] OR ("oceans"[All Fields] AND "seas"[All Fields]) OR "oceans and seas"[All Fields] OR "oceans"[All Fields]) OR "underwater"[All Fields]) | 51 |

Web of Science

All Databases

- WOS: 1900 to 2023
- ARCI: 2015 to 2023
- BCI: 1926 to 2023
- KJD: 1980 to 2023
- MEDLINE: 1950 to 2023
- SCIELO: 2002 to 2023
- ZOOREC: 1864 to 2023

| # | Search Query | Results |
| --- | --- | --- |
| 1 | TS= dental caries | 89510 |
| 2 | TS= fish | 1418162 |
| 3 | AK= Marine Biology | 403 |
| 4 | KP= Eutrophication | 10636 |
| 5 | TS= Seawater | 147114 |
| 6 | TS= Seaweed | 42955 |
| 7 | TS= Fisheries | 500174 |
| 8 | TS= sea | 1015660 |
| 9 | TS= ocean | 984075 |
| 10 | TS= underwater | 160099 |
| 11 | TS= "plastic debris" | 3730 |
| 12 | #2 OR #3 OR #4 OR #5 OR #6 OR #7 OR #8 OR #9 OR #10 OR #11 | 3118890 |
| 13 | #12 AND #1 | 763 |
| 14 | #12 AND #1 and Child (MeSH Headings) | 137 |

Scopus

| #1 | KEY (caries) | 66,007 |
| --- | --- | --- |
|  | TITLE-ABS-KEY ( ( marine OR ocean OR oceans OR sea OR seas OR coast* OR mangrove ) AND ( {water cycle} OR {water cycles} OR {biogeochemical cycle} OR {biogeochemical cycles} OR {oceanic circulation model} OR {oceanic circulation models} OR {oceanic circulation modelling} OR {oceanic circulation modeling} OR {ice-ocean} OR eutrophicat* OR marine OR {coral bleach} OR {coral bleaching} OR {coastal management} OR {coastal habitat} OR {coastal habitats} OR {marine debris} OR {ocean acidification} OR ( acidification AND seawater ) OR {fishery} OR {fisheries} OR {overfishing} OR {sustainable yield} OR {marine protected area} OR {marine protected areas} OR {marine conservation} OR {ecotourism} OR {community based conservation} OR {community-based conservation} OR {marine land slide} OR {marine pollution} OR {nutrient runoff} OR {coastal ecotourism} OR {destructive fishing} OR {local fisheries} OR {artisanal fishers} OR {fisheries rights} OR {species richness} OR {traditional ecological knowledge} OR {small island development states} OR {marine quota} OR {marine economy} OR {marine policy} ) AND NOT ( {paleoclimate} OR {paleoceanography} OR {radiocarbon} OR {genetics} OR {medicine} OR {drug} OR {engineering} OR {aerosol} ) ) | 498,028 |
| #3 | TITLE-ABS-KEY ( ( marine OR ocean OR oceans OR sea OR seas OR coast* OR mangrove ) AND ( {water cycle} OR {water cycles} OR {biogeochemical cycle} OR {biogeochemical cycles} OR {oceanic circulation model} OR {oceanic circulation models} OR {oceanic circulation modelling} OR {oceanic circulation modeling} OR {ice-ocean} OR eutrophicat* OR marine OR {coral bleach} OR {coral bleaching} OR {coastal management} OR {coastal habitat} OR {coastal habitats} OR {marine debris} OR {ocean acidification} OR ( acidification AND seawater ) OR {fishery} OR {fisheries} OR {overfishing} OR {sustainable yield} OR {marine protected area} OR {marine protected areas} OR {marine conservation} OR {ecotourism} OR {community based conservation} OR {community-based conservation} OR {marine land slide} OR {marine pollution} OR {nutrient runoff} OR {coastal ecotourism} OR {destructive fishing} OR {local fisheries} OR {artisanal fishers} OR {fisheries rights} OR {species richness} OR {traditional ecological knowledge} OR {small island development states} OR {marine quota} OR {marine economy} OR {marine policy} ) AND NOT ( {paleoclimate} OR {paleoceanography} OR {radiocarbon} OR {genetics} OR {medicine} OR {drug} OR {engineering} OR {aerosol} ) ) AND KEY ( caries ) | 22 |
